# Effect of transgenic *Leishmania major* expressing *mLLO-Bax-Smac* fusion gene in the apoptosis of the infected macrophages

**DOI:** 10.22038/IJBMS.2021.56960.12701

**Published:** 2021-12

**Authors:** Maryam Aghaei, Hossein Khanahmad, Akram Jalali, Shahrzad Aghaei, Manizheh Narimani, Sayed Mohsen Hosseini, Fatemeh Namdar, Seyed Hossein Hejazi

**Affiliations:** 1 Skin Diseases and Leishmaniasis Research Centre, Isfahan University of Medical Sciences, Isfahan, Iran; 2 Department of Genetics and Molecular Biology, School of Medicine, Isfahan University of Medical Sciences, Isfahan, Iran; 3 Research Center for Molecular Medicine, Hamadan University of Medical Sciences, Hamadan, Iran; 4 Department of Molecular Medicine, School of Advanced Technologies, Shahrekord University of Medical Sciences, Shahrekord, Iran; 5 Department of Parasitology and Mycology, School of Medicine, Isfahan University of Medical Sciences, Isfahan, Iran; 6 Department of Biostatistics & Epidemiology, School of Public Health, Isfahan University of Medical Sciences, Isfahan, Iran; 7 Department of Parasitology and Mycology, School of Medicine, Isfahan University of Medical Sciences, Isfahan, Iran; 8 Skin Diseases and Leishmaniasis Research Center, Department of Parasitology and Mycology, School of Medicine, Isfahan University of Medical Sciences, Isfahan, Iran

**Keywords:** Homologous recombination, Integration, Leishmaniasis, Transfection, Vaccine

## Abstract

**Objective(s):**

Leishmaniasis is a complex infection against which no confirmed vaccine has been reported so far. Transgenic expression of proteins involved in macrophage apoptosis-like BAX through the parasite itself accelerates infected macrophage apoptosis and prevents *Leishmania* differentiation. So, in the present research, the impact of the transgenic *Leishmania major* including *mLLO-BAX-SMAC* proapoptotic proteins was assayed in macrophage apoptosis acceleration.

**Materials and Methods:**

The coding sequence *mLLO-Bax-Smac* was designed and integrated into the pLexyNeo2 plasmid. The designed sequence was inserted under the 18srRNA locus into the *L. major* genome using homologous recombination. Then, *mLLO-BAX-SMAC* expression was studied using the Western blot, and the transgenic parasite pathogenesis was investigated compared with wild-type *L. major in vitro* and also *in vivo*.

**Results:**

Western blot and PCR results approved *mLLO-BAX-SMAC* expression and proper integration of the *mLLO-Bax-Smac* fragment under the 18srRNA locus of *L. major*, respectively. The flow cytometry results revealed faster apoptosis of transgenic *Leishmania*-infected macrophages compared with wild-type parasite-infected macrophages. Also, the mild lesion with the less parasitic burden of the spleen was observed only in transgenic *Leishmania*-infected mice. The delayed progression of leishmaniasis was obtained in transgenic strain-injected mice after challenging with wild-type *Leishmania*.

**Conclusion:**

This study recommended transgenic *L. major* including *mLLO-BAX-SMAC* construct as a pilot model for providing a protective vaccine against leishmaniasis.

## Introduction

One of the complex parasitic diseases in more than 102 countries is leishmaniasis which is caused by various species of *Leishmania*, a single-cellular kinetoplastid parasite (1-3). About 21 species of *Leishmania *parasites cause the main forms of leishmaniasis including Cutaneous, Mucocutaneous, and Visceral (2). *Leishmania major *that causes CL, has a haploid genome (29 -33 Mb in size) (4) including 36 chromosomes and 8272 coding genes involved in the surface glycoconjugates synthesis and pathogen-host interactions like proteolytic enzymes (5). The disease is transmitted by an infected phlebotomine sand-fly when it takes blood and injects mobile promastigotes into the host skin. The tissue macrophages of the host quickly capture the parasite, where *Leishmania* spp. within 12 to 24 hr differentiates into the non-motile amastigote to survive in macrophage phagolysosomes and once the macrophage collapses, released amastigotes infect new macrophages (6).

For disease resolution, macrophages attack *Leishmania* parasites through toxic oxygenated metabolite products such as anion superoxide (O2^-^) and also present the *Leishmania* antigens to the TCD4^+^ cells. TCD4^+^ cell with the production of IFN-γ, stimulates nitric oxide production in infected macrophages for killing the *Leishmania* parasites, effectively (7). In contrast, *Leishmania* amastigotes inhibit macrophage function through down-regulating MHC class II and enhancing the production of the regulatory cytokine-like TGF-β and IL-10 (8). 

Another way for the rapid deletion of *Leishmania *by macrophages is apoptosis (9)*. Leishmania* induces cellular stress responses in macrophages and stress activates JNK and C-JUN/AP-1 signaling pathways (10). As in these signaling pathways, JNK induces c-Jun/AP-1 and enhances the death ligand FasL expression. Thus, cellular responses to stress lead to Fas-mediated apoptosis) the extrinsic apoptotic pathway). In the presence of interferon-γ (IFN-γ), macrophages infected with *L. major* increase the Fas expression and become sensitive to cytotoxic T cells CD4 expressing FasL (11). Also, FasL and IFN-γ enhance the killing of *L. major* by macrophages (12).

The parasites can delay macrophage apoptosis in different ways like activating Extracellular signal-regulated kinases (ERK1/2) (13) and Phosphoinositide 3-kinases/Acetate kinase (PIK3/ACK) pathways and preventing caspase 3 and 7 processes, inhibiting expression of BAX gene, stimulating anti-apoptotic signals, and secretion of cytochrome C (14). 

The *BAX *gene in the 19q13.3-q13.4 of the human genome is known as the first pro-apoptotic protein of the BCL-2 family (15, 16). The main form of BAX (BAX α) with 21 kDa exists at the mitochondrial outer membrane (MOM) for apoptosis induction (15, 16). It is present in the latent form in tissues like the spleen and breast and activates upon apoptosis induction (17). The active BAX interacts with BID and changes conformationally. Then, the oligomerized BAX intercalates into the MOM and opens the voltage-dependent anion channel (VDAC) in mitochondria. Furthermore, it forms an oligomeric pore of the mitochondrial apoptosis-induced channel (MAC) in MOM that results in the lack of mitochondrion membrane potential and extract of apoptogenic agents such as ROS and cytochrome C of the mitochondrial inter-membrane space into the cytosol (18, 19). The Second mitochondrial-derived activator of caspases (SMAC) is also a protein of inter-membrane space of mitochondria that promotes TNF receptor and cytochrome C-dependent activation of apoptosis through prohibiting the impact of Inhibitor of Apoptosis Proteins (IAPs) (20). Afterward, cytochrome C assembles the apoptosome complex by joining to apoptotic peptidase activating factor 1 (APAF1). This complex perpetrates the cell to apoptosis through binding and activating the cascade of caspase (18). 

Though, caspase 3 and BAX expression increase in cells infected with *Leishmania* by tumor suppressor p53 during 24 hr (21), research results show that *Leishmania* prohibits *BAX* homo-oligomerization resulting in impaired translocation of *BAX* to mitochondria (21). Also, apoptosis prevention is partly associated with equilibrium of *BAX* and *BCL-2 *gene expression. Hence, *Leishmania* opposites the macrophages’ apoptosis by up-regulating *BCL-2* and down-regulating *BAX* (22).

The principal purpose of the current drugs like miltefosine is to induce parasite apoptosis (23) which is used after the appearance of the lesion and has side effects like toxicity to other cells (24). Therefore, new molecular approaches are required for the treatment of leishmaniasis (25). One of the late vaccination strategies is generating transgenic *Leishmania*. This haploid parasite can endure homologous recombination easily and hence deleting or integrating genes into *Leishmania* is possible (26). So far, the making of transgenic *Leishmania* has been done in 3 pathways, consisting of removing genes coding pathogenic factors, generating parasites expressing host immune factors, and parasite labeling (through fluorescence and biochemical reporter genes) for *in vitro *and *in vivo* post-infection examination (27).

One of *Leishmania’s* strategies to get away from the immune system of the host is delaying or inhibiting the macrophage apoptosis and also prohibiting pro-apoptotic proteins like BAK and BAX that can promote this natural process in the macrophage. So, the transgenic expression of pro-apoptotic genes through the parasite itself can enhance the percentage of macrophage apoptosis and stimulate less pathology through increased apoptosis of macrophages and without any side effects on the rest of the cells. We hypothesized that expression of *BAX-SMAC* proteins by transgenic *L. major* can be applied as an effective method to find the alternative to leishmanization against leishmaniasis.

As in this research, transgenic *L. major* expressing and secreting pro-apoptotic *BAX* and *SMAC* proteins of the mouse host was created through homologous recombination, and insertion of mentioned genes in the 18srRNA region of the *L. major* genome was assayed. We found that this transgenic organism can reduce disease progression and induce protection versus virulent organisms through increasing infected macrophages’ apoptosis. So, this study suggested a new method for protection of susceptible individuals against the leishmaniasis disease.

## Materials and Methods

***Study area***

This experimental research was done at the Department of Parasitology and Mycology, Isfahan University of Medical Sciences, Isfahan, Iran between 2015 and 2018, using a grant awarded by Research Vice-Presidency of Isfahan University of Medical Sciences, Isfahan, Iran.

***Construction of the recombinant vector***

**Figure 1:**
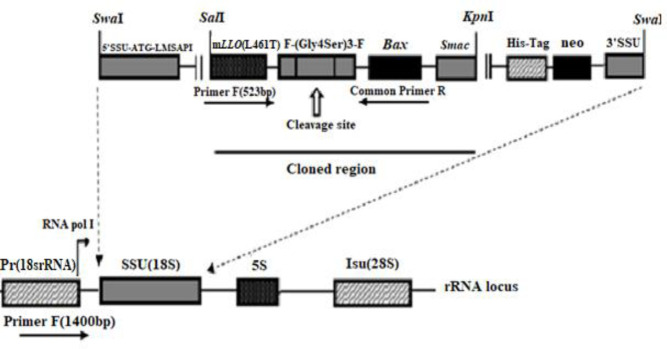
Schematic illustration of the pLEXSY-Neo2-*mLLO-Bax-Smac *expression vector. The *mLLO-Bax-Smac* fragment was subcloned in the pLEXSY-Neo2 vector in *SalI/KpnI* restriction sites. Also, the linearized plasmid with the *SwaI* restriction enzyme will integrate into the 18srRNA region of transfected *Leishmania major* genome by homologs recombination. Forward (F) and reverse (R) primers were shown

The sequence of murine *Bax* *α* gene (AF339055) along with mutated listeriolysin O (m*LLO-*L461T) (LLO lyses the phagosome membrane in pH≤7, while m*LLO* acts in pH≥7) and *Smac *gene (8 a.a) that were placed at the 5ˊ and 3^´′^ ends of the *Bax *gene, were codon-optimized. Moreover, the cleavage site of furin was considered between *Bax-Smac* and m*LLO* sequences. The m*LLO*-*Bax-Smac* sequence with 2292 bp size was ordered from Genecust Company (Luxembourg) for synthesis and cloning into the pUC57 vector in the *Sal*I and *Kpn*I restriction sites. Next, the *Sal*I-m*LLO*-*Bax-Smac*-*Kpn*I fragment was sub-cloned into the identical place in the pLEXSY-Neo2 vector, under the Secreted Acid Phosphatase 1 (SAP1) signal sequence, and was ligated with the T4 DNA ligase enzyme (Thermo Fisher, USA) (Figure 1). Then, the ligated vector was transformed into the competent *E. coli *Top10 (28) and transformed bacteria were selected on ampicillin- Luria- Bertani (LB) agar medium. 

***The extraction of the plasmid***

Transformed bacteria containing pLEXSY-Neo2-m*LLO*-*Bax-Smac *were cultured in ampicillin -LB broth medium,16 hr at 37 ^°^C and 200 rpm (29). The plasmid DNA was extracted using SolGent (Korea). Next, the plasmid was linearized using the *Swa*I restriction enzyme (Thermo Scientific, USA) and separated on a 1% agarose gel electrophoresis. Then, a smaller band including the m*LLO*-*Bax-Smac*+ flank of up and down of the plasmid backbone fragments was extracted from the agarose using a gel extraction kit (Bioneer, Korea).

***The culture of parasite***

*L. major *promastigotes (MRHO/IR/75/ER) were taken from the Department of Parasitology and Mycology, School of Medicine, Isfahan University of Medical Sciences, Isfahan, Iran, and cultured in biphasic NNN medium. Next, the parasites were sub-cultured at 25 ^°^C and in monophasic RPMI 1640 mediums (Gibco, USA) supplemented with 10% fetal bovine serum (FBS) and 100 U/ml penicillin/ streptomycin (Gibco, Pen-Strep15140). 

***Transfection of L. major***

To generate transgenic* L. major*, 1×10^8^ parasites in the logarithmic phase (with OD=2) were harvested and the pellet was washed with cold transfection buffer (4 g NaCl, 2.5 g HEPES [pH 7.5], 0.05 g Na_2_Hpo_4_, 0.594 g/l Glucose, and 0.185 g KCl) two times based on the Jena Bioscience protocol (Jena Bioscience, Germany). Then, the pellet was re-suspended in cold transfection buffer (450 μl) and combined with about 20 µg of extracted vector in a 4 mm cuvette and placed on ice for 10 min. Next, parasite transfection was done with the following conditions: 1600 v, 25 µF, 1.2 ms (30). Also, the wild-type *L. major* was transfected without DNA plasmid (as a negative control). Finally, cuvettes were returned on ice for 10 min and the transfected promastigotes were cultured in the FBS/RPMI1640/Antibiotics (pen/step) liquid media and placed for 48 hr at temperature room. 

***The selection of transgenic promastigotes***

The transfected *L. major* promastigotes were centrifuged and selected in liquid media containing RPMI 1640, 10% FBS, 100 U/ml penicillin/ streptomycin, and 25 µg/ml Geneticin (Roche, Germany), and the selection was followed for two weeks by adding the Geneticin up to 100 μg/ml (30). 

***MTT test***

The growth rate and viability of the transgenic parasites were determined using the MTT test. Briefly, 1×10^6 ^transgenic and wild-type parasites were inoculated in 5 ml of medium) 50:50(. 100 µl culture was harvested daily (up to 7 days) and loaded onto a 96-well plate and 20 µl MTT [3-(4,5-dimethylthiazol-2-yl)-2,5-diphenyltetrazolium bromide] (Sigma) added to wells for 2 hr at 25 ^°^C. Then, to lyse cells, 100 μl of dimethyl sulfoxide (DMSO) was added for 30 min at 25 ^°^C and in the dark condition. Finally, the plate at 550 nm was read using an ELISA plate reader (30). 

***PCRs method***

To confirm the correct insertion of the m*LLO*-*Bax-Smac* cassette into the 18srRNA region of the transfected* L. major* genome by homologous recombination, genomic DNA of transfected promastigotes was extracted using a Genetbio kit (South Korea). Then, long-range PCR was carried out with forward primer hybridizing to the 18srRNA region upstream on *Leishmania* genomic DNA and reverse primer hybridizing to the expression cassette. Also, some parts of the m*LLO*-*Bax-Smac* segment and ITS-1 (Internal Transcribed Spacer 1 gene as a control) were amplified using primer pairs listed in Table1.

***Protein preparation***

The wild type and transgenic *L. major* were cultured in conditioned medium (serum and bicarbonate sodium-free-RPMI 1640 contain 100 U/ml penicillin/streptomycin/gentamicin) at 25 ^°^C for 48 hr. Next, the supernatants were processed in accordance with the Cuervo method with some modification. Briefly, the supernatants were centrifuged in two steps (2000 ×g, 10 minutes, 4 ^°^C and 20,000×g, 1 hr, 4 ^°^C). Then, TCA (trichloroacetic acid) with %10 and %20 concentrations were added, and for 60 min the solutions were incubated on ice. Finally, the pellets were washed with cold acetone at 14000 ×g, 4 ^°^C,15 min, and solved in distilled water (31). 

***SDS-PAGE and Western blotting***

Identical amounts of condensed proteins from both parasites were run on sodium dodecyl sulfate-polyacrylamide gel electrophoresis (SDS-PAGE) 12% and transferred to the nitrocellulose membrane at 80 V and 4 ^°^C for 90 min. Next, the membrane was blocked in skim milk 5% (W/V) for 2 hr at 25 ^°^C and incubated in a diluted mouse anti-(6×His) HRP conjugated antibody (1:1000, Sigma, USA) for 16 hr. Finally, tetramethylbenzidine (TMB) substrate was added to visualize ​ HRP-conjugated IgG bonded to the his-tagged fusion protein (32). 

***Hemolysis test***

To survey the hemolysis by the transgenic *L. major* and wild-type *L. major* (as control), the linear culture was done on the blood agar media including defibrinated sheep blood, nutrient agar, ampicillin, rifampicin, and plates were placed for 7 days at 24-26 ^°^C (33). 

***In vitro assay***

To compare the acceleration of apoptosis in macrophages infected with transgenic and wild–type *L. major*, the J774 cell line was bought from Pasteur Institute of Iran, Tehran, Iran, and cultured in a medium containing RPMI 1640, FBS 10%, 100 U/ml penicillin/streptomycin at 37 ^°^C and 5% CO_2_. Then, macrophages were infected with transgenic and wild-type *L. major* promastigotes in late-stationary-phase, at a cell-to-parasite ratio of 1:10. Afterward, macrophage lysate and supernatants were harvested at 12, 24, and 48 hr post-infection. Next, necrosis and apoptosis percentages of both infected macrophages were assayed using the flow-cytometry method (BD Biosciences kit, Roche) at the mentioned times (34, 35). Also, the 8-hr macrophage culture was fixed with absolute methanol on the slide and stained with Wright-5% Giemsa stain for 3 min and observed by light microscopy (x1,000) (36). This test was performed with triple replications of each well (36).

***In vivo assay***

30 Balb/c mice (inbred females, 4-6 weeks old) were bought from the animal breeding facility of Pasteur Institute of Iran and maintained in a conventional animal facility at Isfahan University of Medical Sciences. This research was verified by the Institutional Review Boards (IRB) of Isfahan University of Medical Sciences with IR.MUI.REC.1394.3.791 ethical approval number.

To assay the pathogenesis of the transgenic parasites, mice were grouped as 10 wild-type-infected mice (group1), 10 transgenic *L. major*-infected mice (group2), and also 10 mice infected with both transgenic and wild-type *L. major *(group 3). Groups 1 and 2 were inoculated with 1×10^5^ stationary phase promastigotes from wild-type and transgenic *L. major* subcutaneously at the tail base, respectively. Furthermore, group 3 was injected with both types of parasites (1×10^3^+1×10^3^). Disease flow was followed using a vernier caliper to measure ulcer size, twice weekly (30). 

***Re-infecting transgenic L. major-infected mice with wild-type L. major***

To evaluate the potency of the transgenic parasites to protect Balb/c mice, un-ulcered mice of 2 and 3 groups (mice infected with transgenic *L. major* and both transgenic and wild-type parasites, respectively) were re-infected with 1×10^5^ wild-type promastigotes after 4 weeks, and the course of the disease was followed across 4 weeks (30). 

***The Parasite burden evaluation***

After four weeks (day 30), the mice in all groups were sacrificed and their spleens were excised. The weighted piece of spleen of each mice (~20 mg) (37) was homogenized in 2 ml of Schneider’s medium (Sigma, Germany) containing gentamicin 0.1% and FBS 20%, in a sterile condition by a tissue grinder. The homogenized tissues were diluted with the same medium from 1 to 10^-10^ in a 96-well plate and maintained for 7 days at 27 ^°^C. Then, the attendance of motile parasites in wells was surveyed using an inverted microscope (40 magnification). The end titer was the dilution with a minimum of one live promastigote. Finally, the parasite burden/mg of tissue was obtained using the below formula:

Parasite burden=-log_10_(parasite dilution/tissue weight) (38)

***Statistical analysis***

Data were shown as means±SEM (standard error of the mean) and calculated using ANOVA, Tukey, and Multivariate tests. Also, SPSS 16 software was used for statistical analysis and the *P*-value<0.05 was considered for significant differences.

## Results

***Cloning of mLLO-Bax-Smac sequence***

In the present study, after subcloning the *mLLO*-*Bax-Smac* fragment in pLEXSY-neo2, the correct integration of sequence was confirmed using *Sal*I/*Kpn*I restriction enzymes, as the results showed two 7911bp and 2292bp fragments of the backbone of the plasmid and *mLLO*-*Bax-Smac* CDS, respectively. Then, we integrated the expression cassette of the *mLLO*-*Bax-Smac* fusion gene of pLEXY-*mLLO-Bax-Smac* into *L. major *genome using transfection through homologous recombination. The pLEXSY-neo2 vector is known as a common vector for transferring the gene in the 18srRNA region of the* Leishmania *genome through 5ʹuss and 3ʹuss and homologous recombination. 18srRNA promoter of *Leishmania* as well as signal peptide of SAP1 of pLEXSY-neo2, were used for expression and secretion of the fusion gene. Also, His-tag (6× his) was embedded at the C- terminal of fragment encoding m*LLO*-*Bax-Smac *(Figure 1).

Furthermore, the gene script webserver was used to detect the CAI of the codon-optimized m*LLO*-*Bax-Smac* CDS, and increasing CAI from 0.58 to 0.91 showed a suitable index of expression.

***Transfection of L. major with pLEXY-mLLO-Bax-Smac plasmid***

**Figure 2:**
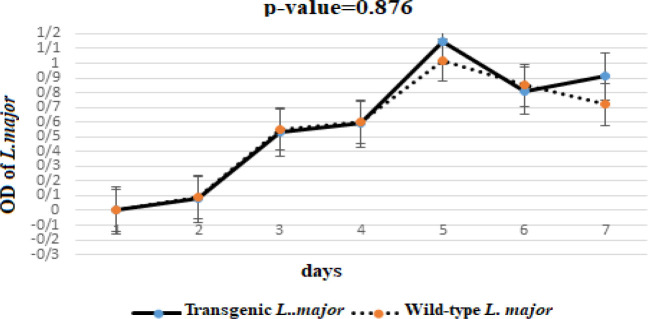
Comparison of growth curves of transgenic and wild-type *Leishmania major* was obtained from the MTT test (mean±standard deviation). Both parasites showed similar growth characteristics

To generate transgenic *Leishmania*, the pLEXY recombinant vector was digested using the* Swa*I restriction enzyme and desired fragment (7880 bp) consisting of pLEXSY-*mLLO*-*Bax-Smac* was extracted from gel. Then, *Leishmania* was successfully transfected with a linear pLEXSY-*mLLO-Bax-Smac* plasmid, as resistant organisms were grown in a liquid medium including Geneticin up to 100 μg/ml concentration. Furthermore, the results of the MTT test revealed the same growth curve of both the transgenic and wild-type parasites, and integration did not show an impact on the metabolic rate and replication of *Leishmania* (*P*= 0.876) (Figure 2).

**Figure 3:**
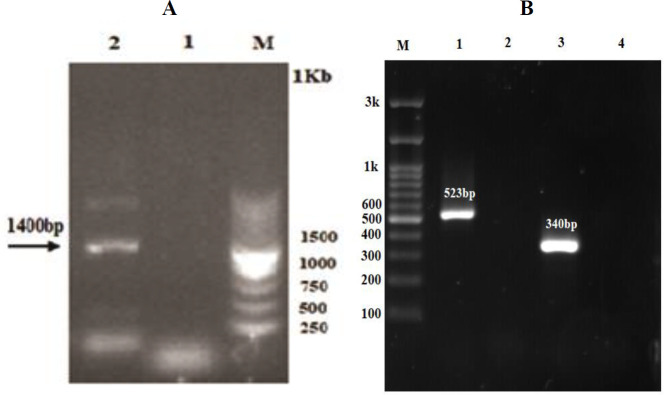
(A): Long-range PCR on extracted genomic DNA of wild-type and transgenic *Leishmania major* using primers hybridizing to upstream of the 18srRNA locus. Lane M: DNA size marker; Lane 1: Wild-type *L. major*; Lane 2: 1.4 kbp PCR product showed fragment integration in transgenic *L. major*. (B): PCR with specific primers for *mLLO-Bax-Smac* construct on transgenic *L. major* genomic DNA. Lane M: DNA size marker; Lane 1: 523 bp PCR product in transgenic *L. major *confirmed both random and homologous integrations. Lane 2: Wild-type parasite; Lane 3: PCR with specific primers for *ITS-1* gene on genomic DNA of wild-type *L. major* (positive control); Lane 4: PCR product without genomic DNA (negative control)

Also, the correct integration of the *mLLO-Bax-Smac* expression cassette into the 18s rRNA ribosomal region of the *L. major* genome was confirmed after DNA extraction and PCRs analysis of the transfected *Leishmania* genome. The 1400pb band in long-range PCR showed upstream of the 18srRNA coding region on genomic DNA and some segments of the *mLLO-Bax-Smac* gene (Figure 3A). Moreover, the PCR analysis using specific primers of the* mLLO-Bax-Smac* sequence showed a 523 bp band that did not exist in control reactions (Figure 3).

***Transgenic parasites express recombinant protein:***

**Figure 4:**
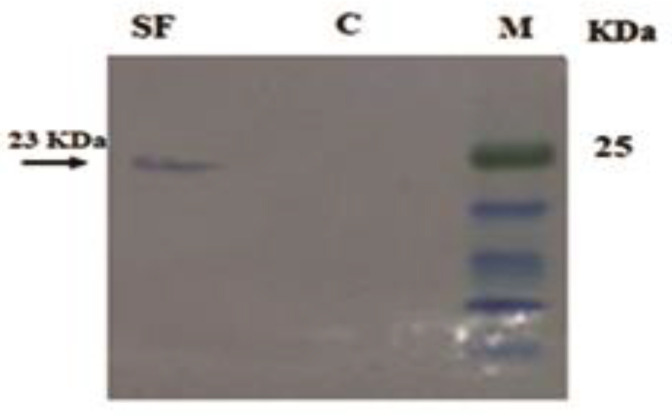
Western blotting analysis of *BAX-SMAC-6-*His fusion protein. (M): Pre-staining protein Marker (SMO431). (C): Secretory protein of wild-type *Leishmania major*. (SF): Condensed secretory fraction (with TCA 20%) of transgenic *L. major*

SDS–PAGE using the secretory protein of transgenic *L. major* showed a band ~23 kDa related to *BAX* fused to *SMAC* compared with the protein of wild-type parasite. Next, the Western blotting result showed a single band ~23 kDa related to the expression of* BAX-SMAC*-6-His fusion protein (Figure 4). 

***Hemolysis test result***

**Figure 5:**
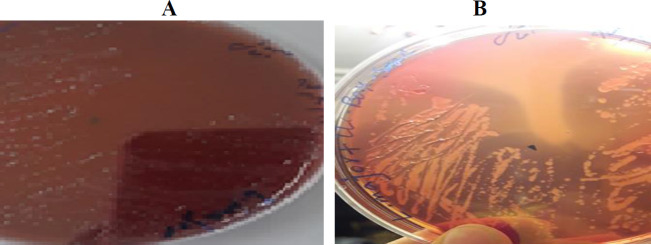
Hemolysis test on the blood agar culture. (A): Hemolysis caused by *LLO* expression in transgenic *Leishmania major*. (B): Absence of hemolysis because of non-expression of *LLO* in wild-type *L. major*

The linear culture results revealed hemolysis on the blood agar plate of transgenic parasites compared with the wild-type *L. major *7 days later (Figure 5).

***In vitro assay results***

**Figure 6:**
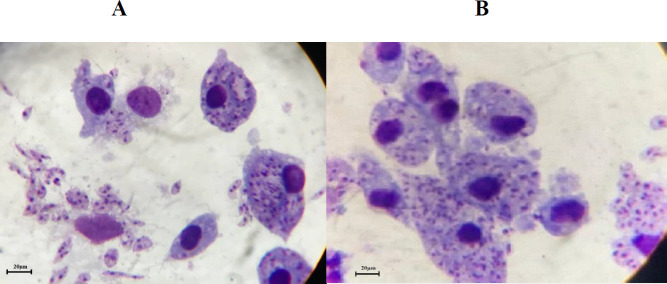
Giemsa staining results of macrophage cells infected with (A): Transgenic *Leishmania major* and (B): wild-type *L. major*, 8 hr post-infection. Both images are at the same magnification×1000. Scale-bar represents 20 µm

**Figure 7:**
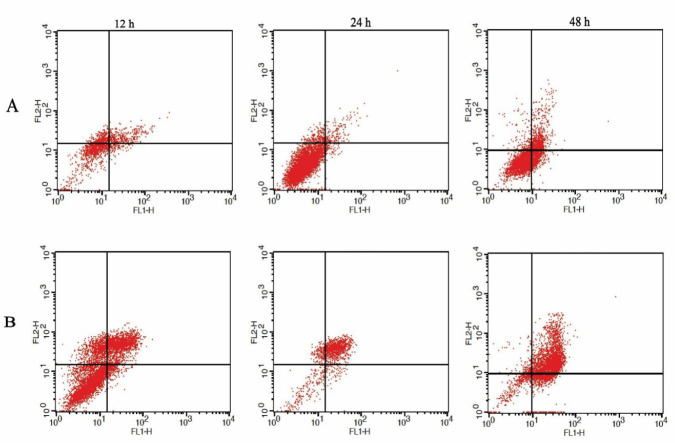
Dot plots of macrophages infected with (A): wild-type and (B): transgenic *Leishmania major* at different times (12, 24, and 48 hr post-infection). FACS plots are gated on macrophage cells. X and Y axes are associated with Annexin V (apoptosis) and PI (necrosis), respectively. Plots show the three separate experiments for each group. Left bottom: viable cells; right bottom: early apoptosis; left top: necrosis; right top: late apoptosis

**Figure 8:**
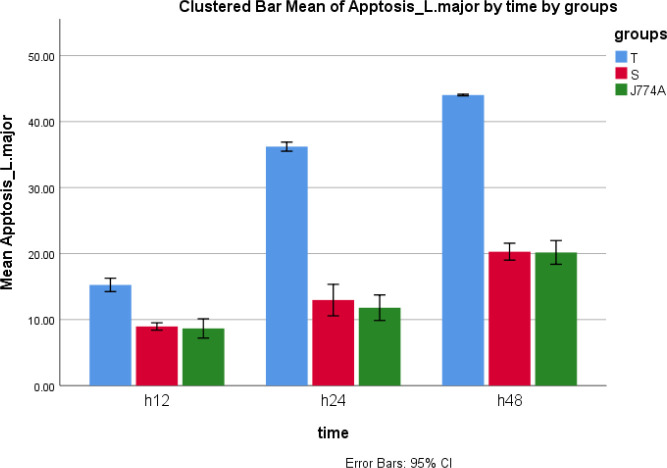
Comparing the mean of apoptosis percentages in the infected macrophages among 3 groups at various times (at 12, 24, and 48 hr). T= Macrophages infected with the transgenic parasite. S= Macrophages infected with wild-type parasite and JJ774A= non-infected macrophages. The macrophages infected with transgenic *Leishmania major* showed increased mean of apoptosis percentage statistically at all times compared with other groups, although there was not a significant difference between macrophages infected with wild-type *L. major* and non-infected macrophages

The Giemsa staining results of macrophages infected with wild-type and transgenic *L. major* parasites showed significant infection of the J744 cells (Figure 6). Furthermore, in statistical analysis of flow cytometry data, the mean of apoptosis percentage among 3 groups was different at 12, 24, and 48 hr (*P*-value<0.000), and revealed accelerated and higher apoptosis rate of macrophages infected with transgenic *L. major* versus the non-infected macrophages and macrophages infected with wild-type *L. major.* There was no significant difference between macrophages infected with wild-type *L. major* and non-infected macrophages at mentioned times (*P*=0.695, 0.207, and 0.958, respectively) (Figures 7 and 8). 

***Less pathology in susceptible BALB/c mice infected with transgenic L. major***

**Figure 9:**
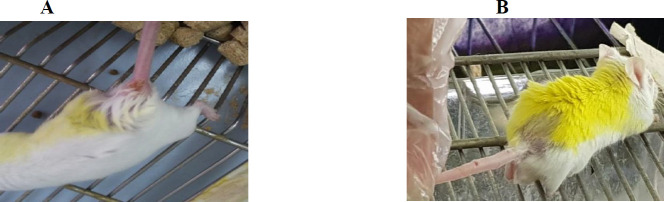
(A): Appearance of the lesion in the tail base of mice infected with wild-type parasite 10 days after injection. (B): lack of lesion or nodule at the tail base of mice infected with transgenic parasite 10 days after injection

**Figure 10:**
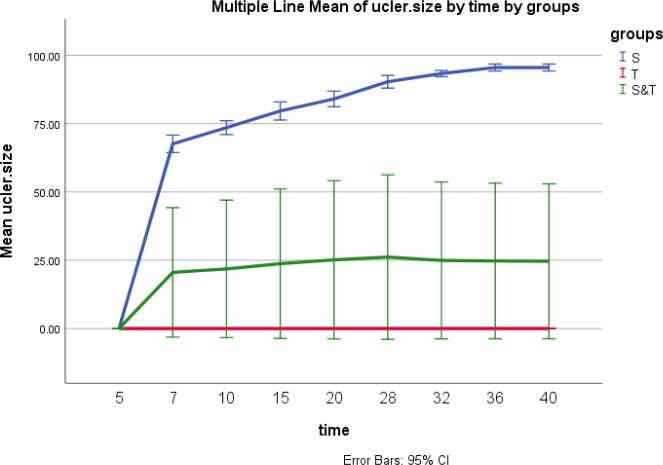
Comparison of the ulcer size among three groups during 40 days. S: mice infected with wild-type parasite. T: mice infected with the transgenic parasite. S&T: mice infected with both wild-type and transgenic parasites. The T group did not show any lesions, and the S &T group revealed smaller lesions than the W group

The appearance of lesions at the tail base of all 10 mice of 1 group during 7-10 days after injection confirmed the CL infection with wild-type parasites (Figure 9A), as the mean of ulcer size increased over time. During 40 days of follow-up, no nodules or lesions were observed in 10 mice injected with transgenic parasites (group 2) (Figure 9B). Also, only 3 mice of group 3 (mice injected with both wild-type and transgenic parasites) revealed small and delayed lesions compared with group 1 (Figure 10). 

***Established protection by transgenic L. major against re-infection with wild-type parasites***

Three weeks after re-infecting with the WT parasites, 3 of the 7 non-ulcered mice of the group injected with both transgenic and wild-type *L. major* (group 3) developed small nodules (without an ulcer), while none of the mice injected with transgenic parasites (group 2) showed lesions or nodules.

***Results of the parasite burden assessment***

**Figure 11:**
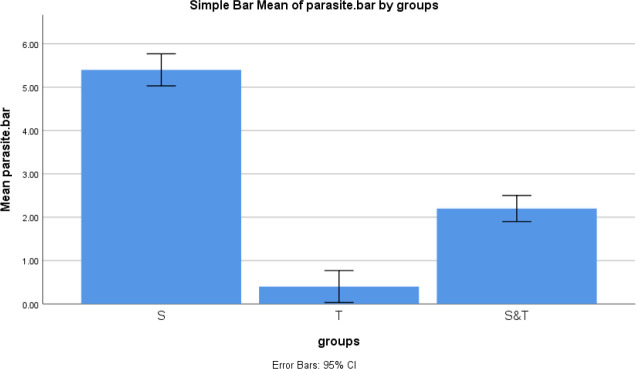
Comparing the mean of spleen parasitic burden among three groups.1: Mice infected with wild-type* Leishmania major*. 2: Mice infected with transgenic *L. major*. 3: Mice infected with transgenic and wild-type *L. major*. Group 2 showed a lower mean of spleen parasitic burden than groups 1 and 3. Furthermore, group 3 showed a lower mean of spleen parasitic burden than group 1

The ANOVA test results showed the different parasitic burdens (*P*-value<0.000) in the spleen of mice infected with the transgenic parasite in comparison with mice infected with wild-type parasite*s *and both wild-type and transgenic parasite. Also, the Tukey test showed a significant difference in spleen parasitic burden between groups 1 and 2 (*P*-value<0.000), groups 1 and 3 (*P*-value<0.000), and groups 2 and 3 (*P*-value<0.000) (Figure 11). 

## Discussion

Over the years, researchers have found that *Leishmania* as an intracellular organism can be resolved from the vertebrate host body exactly via induction of the cellular immune system. In this way, some physiologic properties of the macrophages can be used to accelerate this process. It seems that activation of early apoptosis of macrophages and the endosomal escape of intracellular pathogens like *Leishmania* could be a potent strategy to protect the human host. The first experiment for endosomal escape was performed by Kaufman *et al*. (39, 40) that developed a ∆ureC hly+ rBCG and evaluated the potency and safety of this transgene organism against tuberculosis. 

In this study, the *mLLO*-*Bax-Smac* fusion gene was inserted in *L. major *18srRNA promoter using transfection. Leucine 461 in the *LLO* gene appears to be responsible for its high activity at pH<7. Replacement of non-polar leucine 461 with polar threonine eliminates the functional dependence of this enzyme on pH<7 by creating additional hydrogen bonds and reducing hydrophobicity, so the mutant enzyme can function in both neutral and acidic pH. Also, the cleavage site of furin was embedded to cut *mLLO *from *BAX-SMAC* using host phagosome furin. The designed sequence was codon-optimized based on the codon usage of *L. major*, as CAI (0.91) supported the efficient expression of *mLLo-BAX-SMAC* by *L. major* (41). Moreover, the molecular weight of the designed sequence was estimated at about 84 kDa and the Western blotting detected a band ~23kDa that belonged to *BAX-SMAC-*6×His. It proposes that probably the furin-like proteases have made incisions in the furin cleavage site of the fusion protein and decreased the protein size from 84 to 23 kDa.

Thus, expression of the C-terminal fragment of the fusion protein (*BAX-SMAC*) confirmed the N-terminal fragment expression (*mLLO*). Furthermore, the observed hemolysis of transgenic *L. major *versus wild-type* L. major *confirmed the *mLLO* expression*.*

The appearance of 1400 bp and 523 bp bands using PCR also verified proper replacement of the target sequence into the 18srRNA region of *L. major *via homologous recombination. Field *et al*. (30) also, constructed the transgenic *L. major *expressing the CD40L extracellular portion, and similar to our study, they showed the integration in the 18S rRNA gene did not affect the viability, as the growth curve for transgenic parasite was the same as wild-type *L. major.*

Although, according to Jena Bioscience codon optimization increases expression (42), in this research, the secretory protein concentration of transgenic *L. major* was detected about 1.4 mg/ml which is a relatively low concentration in comparison with previous studies’ amounts like the Kianmehr *et al.* study (29(. However, in another study on the *mLLO*-*Bax-Smac* expression by transgenic *L. infantum*, we found a similar amount (1.5 mg/ml) of protein expression (27) which could be caused by lower copy number of the gene inserted in the host’s genome. Thus, further optimization of expression conditions such as the culture method probably could enhance the recombinant protein production.

Moreover, the flow cytometry results revealed acceleration and increase of apoptosis rate in macrophages infected with transgenic *L. major. *Statistically, a significant difference was seen in the mean of apoptosis percentages of macrophages infected with transgenic parasites versus the wild-type parasite-infected macrophages at 12, 24, and 48 hr. 

According to the hemolysis test results, it can be proposed that the mutated *LLO* protein lyses the endocytic vesicles before developing secondary lysosomes with acidic pH. The study of Glomski *et al.* (43) revealed bacteria expressing natural and mutated LLO could destruct J774A macrophages within 8 and 5 hr post-infection, respectively. So, a significant difference in the apoptosis rate between transgenic and wild-type *L. major* parasites could be because of the secreted mutant LLO of the transgenic strain that demolishes the phagosomal membrane and facilitates the accession of the parasite antigens and phagosomal proteases into the cytosol. 

*SMAC* as an adjuvant to *BAX* protein along with *BAX-SMAC *fusion protein and other lysosomal proteases (like cathepsin) secretes from the phagolysosomes into cytosol which results in caspase activation, internal pathway induction, and enhanced apoptosis of macrophages infected with transgenic* L. major*. Hence, it could be suggested that apoptosis is not only due to *BAX-SMAC* protein expression (40). Similar to our study, Esseiva *et al.* (44) concluded that mammalian BAX expression induces trypanosomatid apoptosis (*T. brucei*), although contrary to our results the events mentioned in their study were temporary and also reversible.

Furthermore, *in vivo* experiments revealed that integration of *mLLO-Bax-Smac *fragment into *L. major* genome affects the biomarkers and capability of the parasite to replicate and also induce infection in sensitive Balb/c mice. Contrary to the mice infected with wild-type parasite which showed lesions at the tail base, no nodules or lesions did appear in the transgenic *L. major*-infected mice. Probably, the suitable function of *mLLO* in perforating the phagosomal membrane and facilitating *BAX-SMAC* transfer into the host cell cytosol causes accelerated and enhanced apoptosis and also apoptotic body formation that finally result in TCD8^+^ and dendritic cell activation, the process referred to as cross-priming (40, 45). So, T cell-associated cellular immunity inhibits parasitic infection and lesion formation by preventing the promastigote differentiation into amastigote that finally led to a loss of nodule or lesion in this group. Also, only 1/3 of mice infected with both wild-type and transgenic *L. major* revealed small and delayed lesions in comparison with mice infected with wild-type *L. major*. Furthermore, the spleen parasitic burden of the control was higher than groups receiving transgenic parasites and transgenic plus wild-type parasites. Similar to our study, researchers (46) by generating transgenic *L. major* expressing murine chemokine monocyte chemoattractant protein 1 (MCP-1), showed that infection of C57BL/6, BALB/c, or MCP-1 knockout (KO) mice with these transgenic *Leishmania* caused small lesions with fewer parasites in the infected foot, spleen, and lymph node versus mice infected with the wild-type. Also, Field *et al*. (30) found that BALB/c mice infected with transgenic *L. major* encoding CD40L presented fewer lesions with lower parasites than animals infected with wild-type *L. major.*

Also, we did not see any lesion or nodule after re-infecting mice protected with the transgenic parasite, whereas after re-infecting mice infected with wild-type plus transgenic *L. major*, small nodules were seen. It showed that *BAX* expressed by transgenic parasites is unable to act with complete efficiency due to the presence of wild-type parasites’ anti-apoptosis mechanisms. In agreement with our results, researchers (47) assayed the immunogenicity in C57BL/6 mice vaccinated with transgenic *L. major* encoding the thymidine kinase gene of Herpes Simplex Virus type 1 (HSV-TK) and cytosine deaminase gene of *Saccharomyces cerevisiae* (Se-cd)), as suicide genes. They concluded that the next re-infect in vaccination prevented disease progression which was due to inducible expression of the suicide genes and high level of immunity. Despite the limitations of this study, including long time and cost, this study is the first to make a transgenic *L. major* that expresses proapoptotic proteins (BAX-SMAC) that could be used as a protective agent or vaccine alternative to leishmanization against CL in endemic areas in the future.

**Table 1:** Primer information used in PCR

Primer	Primer sequence (5ʹ-3ʹ)	Annealing temp.	Amplicon size (bp)
US18rRNAF *LLO-Bax-Smac*-R	TCAAGGACTTAGCCATGCATG CTTGTCAATCTCGTCTGCGTG	60 ^0^C	1400
*LLO*-*Bax-Smac*-F *LLO-Bax-Smac*-R	AATGGATTCGGAACTTTGGTC CTTGTCAATCTCGTCTGCGTG	60 ^0^C	523
ITS-1-F ITS-1-R	CTGGATCATTTTCCGAT TGATACCACTTATCGCACTT	53 ^0^C	340

## Conclusion

In this study, the persistent replacement of the *mLL0-Bax-Smac* gene into the *L. major* genome was done successfully. Also, secretion and expression of recombinant protein were verified and we found that transgenic *L. major* could enhance the macrophage apoptosis rate and induce less pathogenicity and more immunization in comparison with wild-type organisms. So, it seems that these findings can be proposed as an experimental model for creating a protective anti-leishmaniasis vaccine.

## Authors’ Contributions

MA contributed to the conception of the work, conducting the study, drafting and revising the draft, and approval of the final version of the manuscript. HKh contributed to the conception of the work, conducting the study, and approval of the final manuscript. AJ contributed to conducting part of the experiments. ShA contributed to conducting the study, drafting and revising the draft, and approval of the final version of the manuscript. MN contributed to conducting part of the experiments. SMH analyzed study-obtained data. FN contributed to conducting part of the experiments. SHH directed the study and revised and approved the final version of the manuscript. 

## Ethical Approval

Experimental protocols of this study as a PhD thesis (No.394791) were approved by the Institutional Research and Ethics Committee of Medical Sciences, Isfahan University of Medical Science, Isfahan, Iran. The code of ethical approval for the study is IR.MUI.REC.1394.3.791.

## Funding

The study was performed using a grant awarded by the Vice Presidency of Research of Isfahan University of Medical Sciences, Isfahan, Iran.

## Data Access Statement

The data associated with this study are available upon reasonable request. 

## Conflicts of Interest

None declared. 
